# Avatrombopag, an Alternate Treatment Option to Reduce Platelet Transfusions in Patients with Thrombocytopenia and Chronic Liver Disease-Integrated Analyses of 2 Phase 3 Studies

**DOI:** 10.1155/2020/5421632

**Published:** 2020-01-25

**Authors:** Fred Poordad, Norah A. Terrault, Naim Alkhouri, Wei Tian, Lee F. Allen, Mordechai Rabinovitz

**Affiliations:** ^1^Texas Liver Institute and University of Texas Health San Antonio, San Antonio, TX, USA; ^2^Keck Medicine at University of Southern California, Los Angeles, CA, USA; ^3^Texas Liver Institute, San Antonio, TX, USA; ^4^Dova Pharmaceuticals, Inc., Durham, NC, USA; ^5^University of Pittsburgh, Pittsburgh, PA, USA

## Abstract

**Aims:**

Thrombocytopenia complicates the management of patients with chronic liver disease (CLD) undergoing invasive procedures with a bleeding risk. Until recently, prophylactic platelet transfusion was the only treatment option, but has significant safety and efficacy limitations. Phase 3 data demonstrated the superiority of avatrombopag to placebo in reducing platelet transfusions for bleeding, supporting its recent approval.

**Methods:**

Integrated analyses of pooled data (*N* = 435) from two randomized, double-blind, placebo-controlled, phase 3 studies assessed the original efficacy endpoints. Additional analyses included subgroup analyses, alternate Baseline platelet count definitions, and another efficacy endpoint.

**Results:**

Avatrombopag was superior to placebo in increasing patients not requiring a platelet transfusion or rescue procedure, those achieving a platelet count ≥50 × 10^9^/L on Procedure Day, and the change in platelet counts from Baseline. The avatrombopag treatment effect was consistently positive across clinically important disease and Baseline clinical characteristic subgroups, and using alternate Baseline platelet count cohort definitions. Similarly, more avatrombopag-treated patients achieved ≥50 × 10^9^/L platelets with an increase of ≥20 × 10^9^/L from Baseline. The incidence and severity of adverse events were similar between avatrombopag and placebo. Further, safety data demonstrated a low risk for thromboembolic events and hepatotoxicity.

**Conclusion:**

These integrated analyses confirmed the superiority of avatrombopag to placebo in reducing platelet transfusions or rescue procedures for bleeding in patients with thrombocytopenia and CLD scheduled to undergo an invasive procedure, and its tolerable safety profile. Importantly, these data warrant reconsideration of clinical decision making regarding the need to treat thrombocytopenia in patients with CLD. This trial was registered with NCT01972529 and NCT01976104.

## 1. Introduction

Thrombocytopenia (platelet count <150 × 10^9^/L) is common in patients with chronic liver disease (CLD), affecting up to 84% of patients with cirrhosis [1, 2], and worsens with the severity of liver disease; it is associated with increased risks of bleeding, morbidity, and mortality [2–4]. Thrombocytopenia complicates the management of patients with CLD, who require multiple, routine, invasive procedures over the course of their disease, many with a bleeding risk [1, 5]. The risk of bleeding varies with degree of thrombocytopenia, the patient's coagulopathy status and type of procedure [2, 4, 6].

The decision to prophylactically treat thrombocytopenia in these patients before an invasive procedure is based on an assessment of the bleeding risk, coagulation abnormalities, the procedure, and clinical guidelines [7–13]. While several guidelines recommend prophylactic platelet transfusion for platelet counts <50 × 10^9^/L undergoing certain invasive procedures, there is no consensus on the need to treat thrombocytopenia associated with CLD, particularly with low-risk procedures [7–13]. Definitive data on actual bleeding rates with various procedures and platelet counts are lacking, and there remains the inability to *a priori* predict which patients undergoing which procedures will have bleeding.

Until recently, platelet transfusion was the only prophylactic treatment option for thrombocytopenia in patients with CLD undergoing a procedure, and it has significant limitations including variable and transient efficacy, and the risks of transfusion reactions and infections, which may be fatal [14]. Another important consideration is the potential development of antiplatelet antibodies after multiple transfusions, which can render these patients refractory to subsequent platelet transfusions [10]. This can negatively impact patient eligibility for liver transplantation, and creates another challenge for managing these patients who have an increased risk of spontaneous bleeding due to gastric and esophageal varices [10]. Further, platelet-transfusion refractoriness often leads to delayed or cancelled procedures, extends hospitalizations, increases bleeding, and decreases survival [15, 16]. Until recently, the lack of alternatives to platelet transfusions, that eliminate their associated risks, had limited the options for healthcare providers to either transfuse or not transfuse platelets for their patients with CLD-associated thrombocytopenia undergoing procedures. Clinicians had to weigh the risks of using prophylactic platelet transfusions against the uncertain bleeding risks of proceeding with a procedure without treating the thrombocytopenia, and, in the latter case, had to assume some risk of bleeding.

In 2018, avatrombopag (Doptelet®) became the first thrombopoietin (TPO) receptor agonist approved by FDA as an alternative to platelet transfusions for the treatment of thrombocytopenia in patients with CLD scheduled to undergo a procedure [17, 18]; subsequently, a second TPO receptor agonist, lusutrombopag (Mulpleta®), was also approved [19, 20]. Avatrombopag binds to a different site than endogenous TPO on the TPO receptor, and mimics TPO's biologic effects, resulting in increased platelet counts [18, 21]. Efficacy and safety data for avatrombopag in treating thrombocytopenia in patients with CLD have been reported [22, 23]. The phase 3 trials (ADAPT-1 and ADAPT-2) enrolled 435 patients and represent the largest published dataset for TPO receptor agonists in the CLD patient population. The aim of this integrated analysis of the pooled data for avatrombopag from the phase 3 trials was to provide additional safety and efficacy data to guide healthcare providers and explore additional, post-hoc, alternate efficacy, and subgroup analyses.

## 2. Materials and Methods

ADAPT-1 and ADAPT-2 were identically designed, global, randomized, double-blind, placebo-controlled, phase 3 studies using avatrombopag to treat adults with thrombocytopenia associated with CLD. Eligible patients were ≥18 years old with CLD (Model for End-Stage Liver Disease [MELD] score ≤24) and a mean platelet count of <50 × 10^9^/L at Baseline. All patients were to undergo a procedure with a bleeding risk that would require a platelet transfusion unless platelet counts increased from Baseline. Patients were excluded if pregnant; had arterial or venous thrombosis; portal vein blood flow <10 cm/second; World Health Organization (WHO) grade 3 or 4 bleeding; abnormal platelet aggregation; or an active infection requiring antibiotics. All patients provided written informed consent.

Eligible patients were enrolled into 1 of 2 tailored dosing cohorts by their mean platelet count at Baseline, then stratified by procedure bleeding risk (low, moderate, or high) and presence of hepatocellular carcinoma (yes or no). Patients in the *Low Baseline Platelet Count Cohort* (<40 × 10^9^/L) were randomized 2 : 1 to receive 60 mg avatrombopag or placebo once daily with food on days 1 through 5; patients in the *High Baseline Platelet Count Cohort* (40 to <50 × 10^9^/L) were randomized 2 : 1 to receive five daily doses of 40 mg avatrombopag or placebo. Doses were selected based on PK/PD modeling to maximize patients achieving platelet counts ≥50 × 10^9^/L, while limiting patients achieving platelet counts >200 × 10^9^/L. Procedure Day was scheduled 5–8 days after the last dose of the study drug (Days 10–13).

Efficacy endpoints were the same in both phase 3 studies. The primary endpoint was the proportion of patients (Responders) who did not require a platelet transfusion or rescue procedure for bleeding after randomization, and up to 7 days following a scheduled procedure. Secondary endpoints included the proportion of patients achieving the target platelet count (≥50 × 10^9^/L) on Procedure Day, and the change in platelet count from Baseline to Procedure Day. For the integrated analyses, additional post-hoc, alternate efficacy analyses included: analyses of the primary endpoint by various Baseline platelet count subgroups (10 to <20; 20 to <30; 30 to <40; and 40 to <50) and using alternate Baseline platelet count cohort cutoffs (<35 × 10^9^/L; 35 to <50 × 10^9^/L); and the proportion of patients with platelets ≥50 × 10^9^/L on Procedure Day and an increase of ≥20 × 10^9^/L from Baseline.

The pooled Full Analysis Set (FAS) was used for all efficacy analyses and included all randomized patients. The pooled Per Protocol Analysis Set (PPAS) was used for a sensitivity analysis, and included all randomized patients who received the study drug and did not have major protocol violations (e.g., Baseline platelet count >50 × 10^9^/L, no planned platelet transfusion, transfusion before Procedure Day, prohibited con-medications, bleeding at Baseline, no conducted procedure, received different dose or <80% of total planned dose). The Safety Analysis Set (SAS) consisted of patients who received ≥1 dose of the study drug and had ≥1 post-dose safety assessment.

## 3. Results

### 3.1. Patients Demographics and Baseline Characteristics

In the pooled FAS (*N* = 435), demographics and Baseline characteristics were generally similar between cohorts and treatment groups. The *Low Baseline Platelet Count Cohort* (<40 × 10^9^/L) included 251 patients (avatrombopag-160; placebo-91); the *High Baseline Platelet Count Cohort* (40 to <50 × 10^9^/L), 184 patients (avatrombopag-117; placebo-67). Patients were mainly male (65.5%), Caucasian (60.5%), and <65 years old (75.2%) with a mean age of 57.2 years. Patients were from Europe (33.3%), East Asia (32.0%), North America (20.5%), and Rest of the World (14.3%). The distribution of age, sex, ethnicity, race, weight, body mass index, and geographic region was generally balanced across treatment groups. The mean platelet counts at Baseline in both the *Low* (avatrombopag-31.8 × 10^9^/L; placebo-31.6 × 10^9^/L) and *High* (avatrombopag-44.3 × 10^9^/L; placebo-44.7 × 10^9^/L) *Baseline Platelet Count Cohorts* were comparable between the two treatment groups. In the pooled FAS, the distribution of low (60.8%), moderate (17.2%), and high (22.1%) bleeding risk procedures was generally balanced across treatment groups.

### 3.2. Patients Disposition and Study Drug Exposure

**Figure 1:**
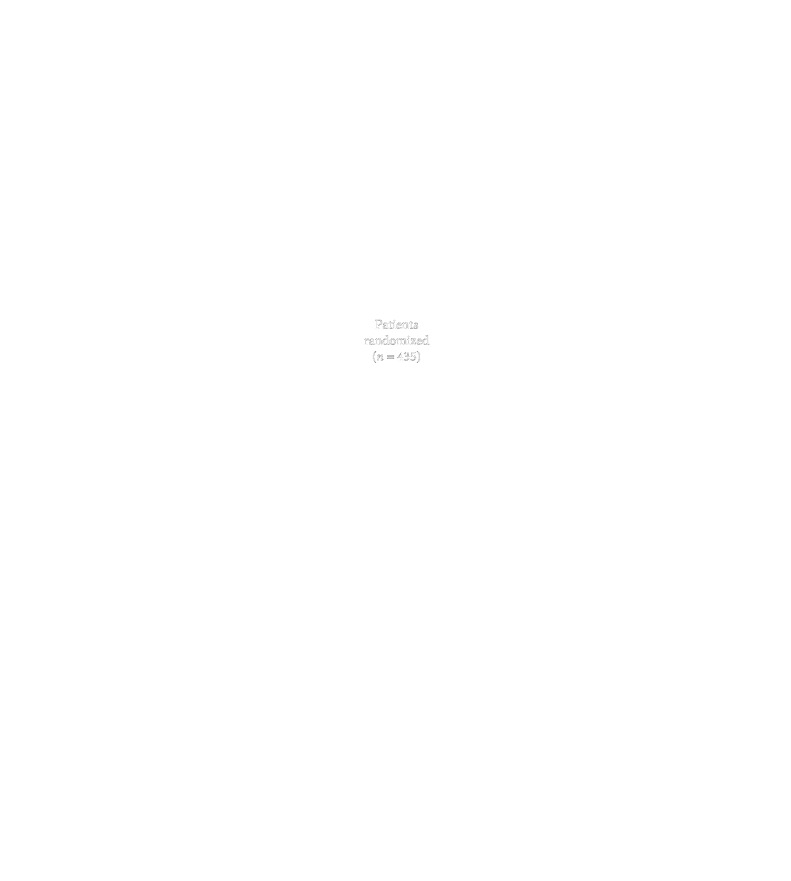
Patients' disposition and primary reason for discontinuation (pooled data from ADAPT-1 and ADAPT-2, all randomized patients). AE = adverse event; EC = entry criteria; LTFU = lost to follow-up; SC = patients' choice; WC = withdrawn consent.

Of the 716 patients screened, 39.2% failed screening because Baseline platelet count was ≥50 × 10^9^/L (14.9%) or portal vein blood flow was <10 cm/second (3.6%). The disposition of randomized patients was similar between treatment groups in both Baseline platelet count cohorts (Figure 1). Only 1 avatrombopag patient (anemia and myalgia) and 1 placebo patient (acute myocardial infarction) were discontinued due to treatment-emergent adverse events (TEAEs). Study drug exposure was comparable across treatment groups in both the *Low* and *High Baseline Platelet Count Cohorts*, with nearly all patients receiving 5 days of treatment (avatrombopag-96.8%; placebo-97.5%).

### 3.3. Efficacy Endpoints

In the integrated analysis of the pooled phase 3 data, avatrombopag was superior to placebo in both Baseline platelet count cohorts in reducing platelet transfusions or rescue procedures, with more Responders to the avatrombopag compared to placebo treatment groups in both the *Low* and *High Baseline Platelet Count Cohorts* with treatment differences that were clinically meaningful and highly statistically significant (Δ38.3%, *P* < 0.0001; and Δ52.2%, *P* < 0.0001, respectively) (Table 1). Nearly all (93.8%) Responders in the avatrombopag treatment group had platelet counts ≥50 × 10^9^/L on Procedure Day, compared to only 38.0% of placebo-treated patients who were “Responders.”

For both Baseline platelet count cohorts, more avatrombopag-treated patients in the pooled FAS achieved the secondary endpoint, achieving a platelet count of ≥50 × 10^9^/L on Procedure Day, compared to placebo, with clinically meaningful and statistically significant treatment differences (*Low Baseline Platelet Count Cohort*: Δ62.6%, *P* < 0.0001; *High Baseline Platelet Count Cohort*: Δ60.7%, *P* < 0.0001 (Table 1)). Importantly, the tailored dosing strategy based on Baseline platelet counts also limited the number of avatrombopag-treated patients achieving platelet counts >200 × 10^9^/L to 3/277 (1.1%).

Similarly, the increase in platelet counts from Baseline to Procedure Day, the second (secondary endpoint) was higher for avatrombopag-compared to placebo-treated patients in both Baseline platelet count cohorts, with statistically significant treatment differences (*Low Baseline Platelet Count Cohort*: Δ26.5 × 10^9^/L, *P* < 0.0001; *High Baseline Platelet Count Cohort*: Δ34.5 × 10^9^/L, *P* < 0.0001 (Table 1)) for both Baseline platelet count cohorts, mean platelet counts in avatrombopag-treated patients approximately doubled from Baseline values.

### 3.4. Primary Efficacy Endpoint Subgroup Analyses

**Figure 2:**
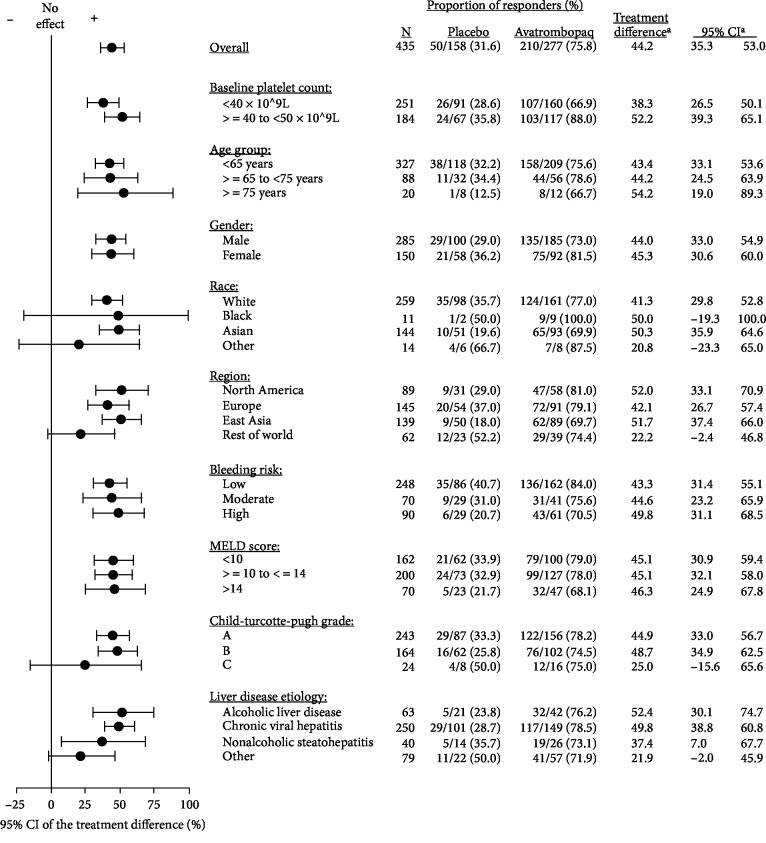
Forest plot of proportion of patients not requiring a platelet transfusion or any rescue procedure, combined Baseline platelet count cohorts—pooled data from ADAPT-1 and ADAPT-2 (full analysis set). CI = confidence interval, MELD = model for end-stage liver disease. ^a^Treatment difference = proportion of responders for avatrombopag−proportion of responders for placebo; 95% confidence interval is calculated based on normal approximation.

A consistently positive avatrombopag treatment effect for the primary endpoint was confirmed across subgroups, including Baseline platelet count, age, gender, race, geographic region, bleeding risk, MELD Score, CTP Grade, and liver disease etiology (Figure 2). The treatment differences were generally similar favoring avatrombopag and were consistent with the results of the overall FAS analysis; subgroups with the smallest number of patients had the widest confidence intervals.

The investigators also identified 110 patients with splenomegaly and 325 patients without. Efficacy endpoints did not differ amongst the 2 populations (data not shown).

### 3.5. Exploratory Analyses

**Figure 3:**
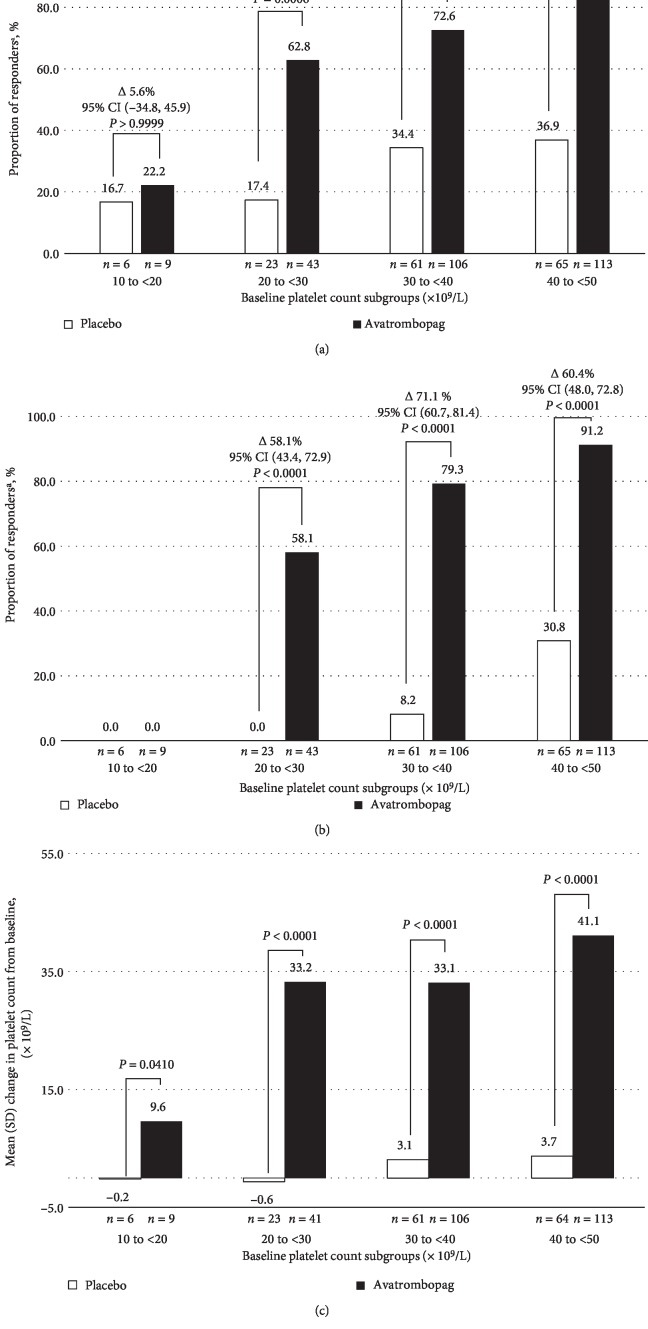
(a) Proportion of patients who did not require a platelet transfusion or rescue for bleeding 7 days post-procedure by individual Baseline platelet count subgroup—pooled data from ADAPT-1 and ADAPT-2 (full analysis set). ^a^Responders were defined as patients who did not require a platelet transfusion or any rescue procedure for bleeding after randomization and up to 7 days following a scheduled procedure. *Note*: *P*-values are based on Fisher's exact test. (b) Proportion of patients achieving the target platelet count of ≥50 × 10^9^/L on Procedure Day by individual Baseline platelet count subgroup—pooled data from ADAPT-1 and ADAPT-2. ^a^Responders were defined as proportion of patients achieving the target platelet count of ≥50 × 10^9^/L on Procedure Day. *Note*: *P*-values are based on Fisher's exact test. (c) Mean change in platelet count from Baseline to Procedure Day by individual Baseline platelet count subgroup—pooled data from ADAPT-1 and ADAPT-2. *Note*: *P*-values are based on Wilcoxon rank-sum test.

In the post-hoc integrated analysis of the primary efficacy endpoint, the proportion of Responders was consistently higher for avatrombopag-treated patients across various Baseline platelet count subgroups (20 to <30, 30 to <40, and 40 to <50 × 10^9^/L), although the difference in the 10 to <20 × 10^9^/L  subgroup (*n* = 15) was small (Figure 3(a)). Overall, there was generally a doubling of platelet counts observed with avatrombopag from Baseline. More patients treated with avatrombopag achieved the secondary endpoint, a platelet count ≥50 × 10^9^/L on Procedure Day, in the 20 to <30, 30 to <40, and 40 to <50 × 10^9^/L Baseline platelet count subgroups compared to placebo (Figure 3(b)). Similarly, across all Baseline platelet count subgroups, the mean change in platelet count from Baseline to Procedure Day was considerably higher in the avatrombopag treatment group, compared to placebo, ranging from approximately 10 to 41 × 10^9^/L (Figure 3(c)).

**Figure 4:**
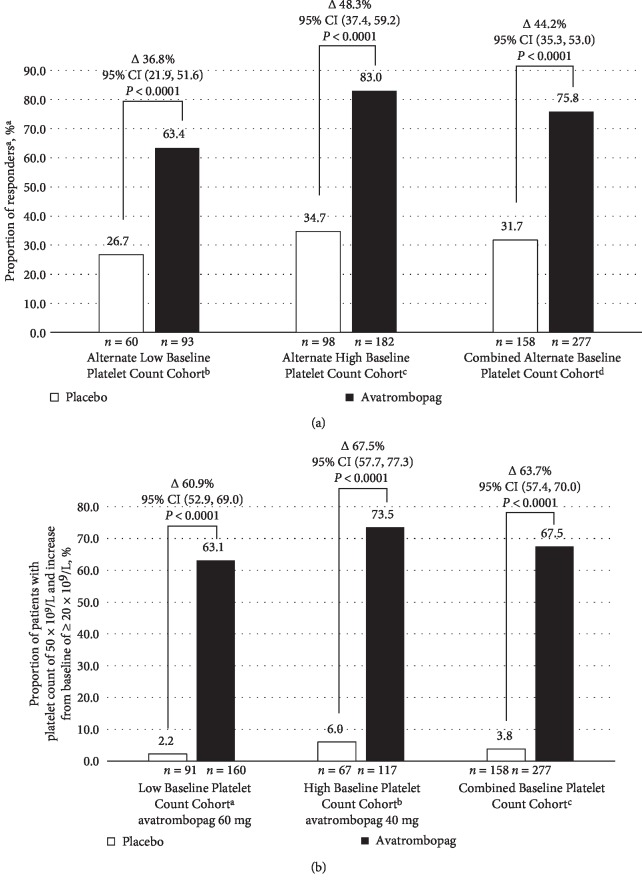
(a) *Alternate Baseline Platelet Count Cohorts*—Proportion of patients not requiring a platelet transfusion or rescue procedure for bleeding—Pooled data from ADAPT-1 and ADAPT-2 (Full analysis set). ^a^Responders were defined as patients not requiring a platelet transfusion or any rescue procedure for bleeding after randomization and up to 7 days following a scheduled procedure. ^b^Alternate Low Baseline Platelet Count Cohort included patients with a platelet count of <35 × 10^9^/L. ^c^Alternate High Baseline Platelet Count Cohort included patients with a platelet count of 35 to ≤50 × 10^9^/L. ^d^Combined Baseline Platelet Count Cohort included patients with a platelet count of <50 × 10^9^/L. ∆ value represents the difference of proportion versus placebo = proportion of Responders for avatrombopag minus the proportion of Responders for placebo. *P*-value is based on Cochran–Mantel–Haenszel Test stratified by the risk of bleeding associated with the scheduled procedure. CI = confidence interval. (b) *Alternate secondary efficacy endpoint analysis*—Summary of proportion of patients that achieved platelet count ≥50 × 10^9^/L and an increase of ≥20 × 10^9^/L on Procedure Day—Pooled data from ADAPT-1 and ADAPT-2 (Full Analysis Set). ^a^*Low Baseline Platelet Count Cohort* included patients with a platelet count of <40 × 10^9^/L. ^b^*High Baseline Platelet Count Cohort* included patients with a platelet count of ≥40 to <50 × 10^9^/L. ^c^Combined Baseline Platelet Count Cohort included patients with a platelet count of <50 × 10^9^/L. ∆ value represents the difference of proportion versus placebo = proportion for avatrombopag minus the proportion for placebo. 95% CI is calculated based on normal approximation. *P*-value is based on Cochran–Mantel–Haenszel test stratified by the risk of bleeding associated with the scheduled procedure. CI = confidence interval.

In the primary endpoint analyses using the alternate Baseline platelet count cohort definition (<35 × 10^9^/L and 35 to <50 × 10^9^/L), avatrombopag was again effective in both cohorts with significantly more Responders in the avatrombopag compared to placebo treatment groups (*Alternate Low Baseline Platelet Count Cohort*: Δ36.8%, *P* < 0.0001; *Alternate High Baseline Platelet Count Cohort*: Δ48.3%, *P* < 0.0001) (Figure 4(a)).

Avatrombopag was also superior to placebo using the alternate secondary efficacy endpoint definition, the proportion of patients with platelet counts ≥50 × 10^9^/L on Procedure Day and an increase of ≥20 × 10^9^/L from Baseline (*Low Baseline Platelet Count Cohort*: Δ60.9%, *P* < 0.0001; *High Baseline Platelet Count Cohort*: Δ67.5%, *P* < 0.0001) (Figure 4(b)).

### 3.6. Safety Analyses

The pooled SAS included 430 patients. The overall incidence of TEAEs was comparable between the avatrombopag and placebo treatment groups (*Low Baseline Platelet Count Cohort*: avatrombopag-56.0%; placebo-58.2%; *High Baseline Platelet Count Cohort*: avatrombopag-51.3%; placebo-50.8%), with most events being mild to moderate (Table 2); the most common TEAEs in both treatment groups included pyrexia, abdominal pain, nausea, and headache. The incidence of treatment-related TEAEs was lower in the combined avatrombopag treatment group (9.5%) compared to placebo-treated patients (12.8%). The incidence of CTCAE Grade 3 TEAEs was similar in the avatrombopag (10.9%) and placebo (10.3%) treatment groups, as was serious TEAEs (avatrombopag-7.3%; placebo-9.0%); the only serious TEAEs reported in more than an individual avatrombopag-treated patient were gastrointestinal hemorrhage and hyponatremia (2 patients each). One avatrombopag-treated patient had a TEAE of partial portal vein thrombosis, and 2 placebo-treated patients had thromboembolic events (acute myocardial infarction; disseminated intravascular coagulation/pulmonary embolus).

## 4. Discussion

The pooled data from the two, identically designed phase 3 studies of avatrombopag in patients with thrombocytopenia and CLD undergoing scheduled procedures, ADAPT-1 and ADAPT-2, provide a robust database (*N* = 435) to further assess the safety and efficacy of avatrombopag, and enabled the evaluation of additional, important post-hoc, alternate efficacy and subgroup analyses to further guide healthcare providers. As previously reported [22, 23], patient demographics and Baseline characteristics were generally well balanced across treatment groups in each study.

Primary endpoint analysis of the pooled ADAPT-1 and ADAPT-2 data confirmed the superiority of avatrombopag to placebo in both Baseline platelet count cohorts, with a higher proportion of avatrombopag-treated patients not requiring a platelet transfusion or rescue procedure for bleeding. The treatment differences were both clinically meaningful and statistically significant (*P* < 0.0001).

Importantly, integrated analyses of the pooled phase 3 data enabled a more robust evaluation of efficacy in various predefined, clinically relevant patient subgroups. The analyses by major demographic factors and relevant intrinsic and extrinsic factors were generally consistent with the overall results in the pooled efficacy analyses of the phase 3 studies. The proportion of Responders in all evaluated subgroups favored avatrombopag, supporting the consistent efficacy of avatrombopag across age, gender, race, region, and procedure bleeding risk. Similarly, efficacy was consistent across key Baseline disease variables, including MELD Score, CTP Grade, and Liver Disease Etiology.

Importantly, the integrated analyses confirmed the rationale for defining tailored dosing based on the platelet count at Baseline, recognizing that patients with lower platelet counts needed a larger increase to reach the target (≥50 × 10^9^/L), and therefore required a higher avatrombopag dose (60 mg). Further from a safety perspective, tailored dosing minimized the number of avatrombopag-treated patients achieving platelet counts >200 × 10^9^/L (1.1%); such high platelet counts have been associated with an increased risk of PVTs with another TPO receptor agonist (eltrombopag) in the same patient population [24]. A further post-hoc analysis of various Baseline platelet count subgroups using the pooled study data showed a consistently higher proportion Responders in avatrombopag-treated patients with counts 20 to <30, 30 to <40, and 40 to <50 × 10^9^/L. While a smaller treatment difference was noted in the smallest Baseline platelet count subgroup (10 to <20 × 10^9^/L; *n* = 15), it remains to be evaluated whether this subgroup would have benefited from a higher dose of avatrombopag.

Efficacy data for another TPO receptor agonist, lusutrombopag, were recently presented using alternate definitions for the Baseline platelet count cohorts and secondary efficacy endpoint [25]. To enable benchmarking, efficacy analyses were conducted with the pooled phase 3 data using these alternate definitions. Again, avatrombopag was superior to placebo in both the *Alternate Low* and *Alternate High Baseline Platelet Count Cohorts*, with more Responders in avatrombopag-treated patients (*P* < 0.0001).

The integrated analyses of both predefined secondary efficacy endpoints using the pooled phase 3 data were consistent with the primary endpoint data, again demonstrating the superiority of avatrombopag over placebo in this patient population. The significantly higher proportion of avatrombopag-treated patients achieving a platelet count of ≥50 × 10^9^/L in both Baseline platelet count cohorts (*P* < 0.0001) is an important clinical endpoint that helps guide clinical decision making regarding the use of avatrombopag. Similarly, there was a significant treatment difference between avatrombopag- and placebo-treated patients for the alternate secondary efficacy endpoint, i.e., the proportion of patients with a platelet count ≥50 × 10^9^/L and an increase of ≥20 × 10^9^/L from Baseline (*P* < 0.0001). Further, the integrated analyses also confirmed the superiority of avatrombopag to placebo for the second, predefined, secondary efficacy endpoint (*P* < 0.0001), demonstrating a larger change in platelet count from Baseline, with an approximately doubling of Baseline platelet counts in avatrombopag-treated patients in both Baseline platelet count cohorts.

Avatrombopag was also shown to be well tolerated in the integrated safety analyses. These safety data support a profile for avatrombopag that was generally comparable to placebo, with the frequency, severity, and types of AEs reported being consistent with those expected in patients with CLD, and no new or unexpected safety signals. There were no safety data to suggest dose-related toxicities, hepatotoxicity, an increased incidence of thromboembolic or bleeding events in avatrombopag-treated subjects, and no rebound thrombocytopenia with discontinuation. Importantly, the recommended dosing regimen of avatrombopag led to a lesser risk for higher platelet counts (≥200 × 10^9^/L) which subsequently translated to a lower risk of thromboembolic events. These data support the use of avatrombopag as a safe alternative treatment option for thrombocytopenia in patients with CLD, and with its recent FDA approval, importantly impact the benefit-risk assessment for the treatment of these patients undergoing scheduled procedures.

Clinical decision making regarding the need to treat thrombocytopenia in patients with CLD remains limited by the lack of definitive data defining the actual risk of bleeding for this patient population, which is further confounded by the variable balance of procoagulant and anticoagulant factors in individual patients, and the differing bleeding risks of specific invasive procedures. For the healthcare provider who has made the decision to treat thrombocytopenia in a specific patient with CLD in advance of an invasive procedure, these safety and efficacy data warrant a reassessment of the benefit-risk profile for using a platelet transfusion versus this new treatment option. Given that avatrombopag is an oral agent that stimulates patients' own megakaryocytes to produce platelets, it eliminates the risks of transfusion reactions or infections, and avoids the development of platelet refractoriness; this importantly preserves the option to utilize platelet transfusions emergently for spontaneous bleeding for which these patients are already at increased risk.

In the absence of compelling data on bleeding risk and globally accepted clinical guidelines for treating these patients undergoing invasive procedures, there remains uncertainty regarding the need to prophylactically treat thrombocytopenia in some cases. The limited utility of routine laboratory-based coagulation tests and their questionable relevance to actual thrombotic and bleeding risks is well established in patients with CLD, and rarely is a comprehensive assessment of individual coagulation factors conducted. Clinicians have been handicapped, having no *a priori* way to predict which patients is undergoing which procedure will continue to have a bleeding complication. Clinical decision making prior to the availability of avatrombopag involved the benefit-risk assessment of platelet transfusion versus no transfusion for the management of thrombocytopenia in patients with CLD undergoing a scheduled procedure. The known risks associated with platelet transfusions and uncertain risks particularly for “low bleeding risk procedures” supported the decision to not transfuse platelets in many cases, accepting that there remained some bleeding risk in doing the procedure without a platelet transfusion. However, with the recent approval of avatrombopag for the treatment of thrombocytopenia in patients with CLD undergoing a scheduled procedure, there are now 3 treatment options to consider: (1) transfuse platelets, (2) not transfuse platelets, or (3) treat with the TPO receptor agonist avatrombopag. The safety and efficacy data for avatrombopag from this integrated analysis of the pooled phase 3 data support a change in the benefit-risk assessment and clinical decision making favoring the use of avatrombopag, and have the potential to change the standard of care for managing thrombocytopenia in these patients with CLD undergoing a scheduled procedure.

## Figures and Tables

**Table 1:** Efficacy analyses—pooled data from ADAPT-1 and ADAPT-2 (full analysis set).

Category	Low Baseline Platelet Count Cohort (<40 × 10^9^/L)		High Baseline Platelet Count Cohort (≥40 to <50 × 10^9^/L)		Combined Baseline Platelet Count Cohort (<50 × 10^9^/L)	
	Placebo	Avatrombopag 60 mg	Placebo	Avatrombopag 40 mg	Placebo	Avatrombopag
*Proportion of subjects not requiring a platelet transfusion or rescue procedure for bleeding*						
*n*	91	160	67	117	158	277
Responders^†^	26 (28.6)	107 (66.9)	24 (35.8)	103 (88.0)	50 (31.7)	210 (75.8)
95% CI	(19.3, 37.9)	(59.6, 74.2)	(24.3, 47.3)	(82.2, 93.9)	(24.4, 38.9)	(70.8, 80.9)
*P*-value^∗^	<0.0001		<0.0001		<0.0001	

*Proportion of subjects who achieved a platelet count ≥50 × 10^9^/L on Procedure Day*						
*n*	91	160	67	117	158	277
Responders^‡^	5 (5.5)	109 (68.1)	20 (29.9)	106 (90.6)	25 (15.8)	215 (77.6)
95% CI	(0.8, 10.2)	(60.9, 75.3)	(18.9, 40.8)	(85.3, 95.9)	(10.1, 21.5)	(72.7, 82.4)
*P*-value^∗^	<0.0001		<0.0001		<0.0001	

*Change in platelet count from Baseline to Procedure Day^§^*						
*n*	91	157	65	116	156	273
Mean (SD) × 10^9^/L	1.8 (8.31)	31.7 (24.83)	3.5 (12.60)	41.0 (30.43)	2.5 (10.31)	35.6 (27.68)
Median × 10^9^/L	0.5	28.0	0.5	37.8	0.5	31.0
*P*-value^∗∗^	<0.0001		<0.0001		<0.0001	

CI = Confidence interval, Max = maximum, Min = minimum, SD = standard deviation. ^†^Responders are defined as the subjects not requiring a platelet transfusion or any rescue procedure for bleeding after randomization and up to 7 days following a scheduled procedure; two-sided 95% confidence interval based on normal approximation. ^∗^*P*-value is based on Cochran–Mantel–Haenszel Test stratified by the risk of bleeding associated with the scheduled procedure within each Baseline platelet count cohort. ^‡^Responders are defined as the subjects who achieved platelet count ≥50 × 10^9^/L on the Procedure Day. ^§^Last-observation-carried-forward is used for subjects with missing platelet count on the Procedure Day. ^∗∗^*P*-value is based on Wilcoxon Rank Sum Test for each avatrombopag treatment group versus placebo within each Baseline platelet count cohort.

**Table 2:** Treatment-emergent adverse events—pooled data from ADAPT-1 and ADAPT-2 (safety analysis set).

Category	Low Baseline Platelet Count Cohort (<40 × 10^9^/L)		High Baseline Platelet Count Cohort (≥40 to <50 × 10^9^/L)		Combined Baseline Platelet Count Cohort (<50 × 10^9^/L)	
	Placebo (*n* = 91)	Avatrombopag 60 mg (*n* = 159)	Placebo (*n* = 65)	Avatrombopag 40 mg (*n* = 115)	Placebo (*n* = 156)	Avatrombopag 40 mg (*n* = 274)
Any TEAE, *n* (%)	**53 (58.2)**	**89 (56.0)**	**33 (50.8)**	**59 (51.3)**	**86 (55.1)**	**148 (54.0)**
Treatment-related TEAEs, *n* (%)	16 (17.6)	18 (11.3)	4 (6.2)	8 (7.0)	20 (12.8)	26 (9.5)
CTCAE grade 3 TEAEs, *n* (%)	12 (13.2)	13 (8.2)	4 (6.2)	17 (14.8)	16 (10.3)	30 (10.9)
Serious TEAEs, *n* (%)	12 (13.2)	11 (6.9)	2 (3.1)	9 (7.8)	14 (9.0)	20 (7.3)
TEAEs leading to study drug withdrawal, *n* (%)	0	2 (1.3)	0	0	0	2 (0.7)
Most frequently reported TEAEs (≥5%), *n* (%)						
Pyrexia (fever)	8 (8.8)	18 (11.3)	6 (9.2)	9 (7.8)	14 (9.0)	27 (9.9)
Abdominal pain	6 (6.6)	10 (6.3)	4 (6.2)	8 (7.0)	10 (6.4)	18 (6.6)
Nausea	7 (7.7)	10 (6.3)	4 (6.2)	8 (7.0)	11 (7.1)	18 (6.6)
Headache	7 (7.7)	7 (4.4)	3 (4.6)	8 (7.0)	10 (6.4)	15 (5.5)
Abdominal pain upper	5 (5.5)	6 (3.8)	3 (4.6)	2 (1.7)	8 (5.1)	8 (2.9)
Procedural pain	2 (2.2)	8 (5.0)	0	0	2 (1.3)	8 (2.9)

TEAE = treatment-emergent adverse event; CTCAE = Common Terminology Criteria for Adverse Events. A TEAE is defined as an adverse event that started on or after the date of first dose of study drug, up to 30 days after the last dose of study drug.

## Data Availability

The data supporting these integrated and subgroup analyses are from previously reported studies, which have been cited.

## References

[1] Maan R., Knegt R. J., Veldt B. J. (2015). Management of thrombocytopenia in chronic liver disease: focus on pharmacotherapeutic strategies. *Drugs*.

[2] Giannini E. G., Greco A., Marenco S., Andorno E., Valente U., Savarino V. (2010). Incidence of bleeding following invasive procedures in patients with thrombocytopenia and advanced liver disease. *Clinical Gastroenterology and Hepatology*.

[3] Mitchell O., Felman D. M., Diakow M., Sigal S. H. (2016). The pathophysiology of thrombocytopenia in chronic liver disease. *Hepatic Medicine: Evidence and Research*.

[4] Afdhal N., McHutchison J., Brown R. (2008). Thrombocytopenia associated with chronic liver disease. *Journal of Hepatology*.

[5] Qureshi K., Patel S., Meillier A. (2016). The use of thrombopoietin receptor agonists for correction of thrombocytopenia prior to elective procedures in chronic liver diseases: review of current evidence. *International Journal of Hepatology*.

[6] Poordad F. (2007). Review article: thrombocytopenia in chronic liver disease. *Alimentary Pharmacology & Therapeutics*.

[7] Rockey D. C., Caldwell S. H., Goodman Z. D., Nelson R. C., Smith A. D., American Association for the Study of Liver Diseases (2009). Liver biopsy. *Hepatology*.

[8] Patel I. J., Davidson J. C., Nikolic B. (2012). Consensus guidelines for periprocedural management of coagulation status and hemostasis risk in percutaneous image-guided interventions. *Journal of Vascular and Interventional Radiology*.

[9] Schiffer C. A., Bohlke K., Delaney M. (2018). Platelet transfusion for patients with cancer: american society of clinical oncology clinical practice guideline update. *Journal of Clinical Oncology*.

[10] Slichter S. J. (2007). Evidence-based platelet transfusion guidelines. *Hematology American Society of Hematology Education Program*.

[11] Szczepiorkowski Z. M., Dunbar N. M. (2013). Transfusion guidelines: when to transfuse. *Hematology American Society of Hematology Education Program*.

[12] Estcourt L. J., Birchall J., Allard S. (2017). Guidelines for the use of platelet transfusions. *British Journal of Haematology*.

[13] ASGE Standards of Practice Committee, Maple J. T., Ben-Menachem T. (2010). The role of endoscopy in the evaluation of suspected choledocholithiasis. *Gastrointestinal Endoscopy*.

[14] Blumberg N., Heal J. M., Phillips G. L. (2010). Platelet transfusions: trigger, dose, benefits, and risks. *F1000 Medicine Reports*.

[15] Stanworth S. J., Navarrete C., Estcourt L., Marsh J. (2015). Platelet refractoriness–practical approaches and ongoing dilemmas in patient management. *British Journal of Haematology*.

[16] Meehan K. R., Matias C. O., Rathore S. S. (2000). Platelet transfusions: utilization and associated costs in a tertiary care hospital. *American Journal of Hematology*.

[17] Shirley M. (2018). Avatrombopag: first global approval. *Drugs*.

[18] Doptlet [package insert] (2018).

[19] Mulpleta [package insert] (2018).

[20] Kim E. S. (2016). Lusutrombopag: first global approval. *Drugs*.

[21] Fukushima-Shintani M., Suzuki K., Iwatsuki Y. (2009). AKR-501 (YM477) a novel orally-active thrombopoietin receptor agonist. *European Journal of Haematology*.

[22] Terrault N., Chen Y. C., Izumi N. (2018). Avatrombopag before procedures reduces need for platelet transfusion in patients with chronic liver disease and thrombocytopenia. *Gastroenterology*.

[23] Terrault N. A., Hassanein T., Howell C. D. (2014). Phase II study of avatrombopag in thrombocytopenic patients with cirrhosis undergoing an elective procedure. *Journal of Hepatology*.

[24] Afdhal N. H., Giannini E. G., Tayyab G. (2012). Eltrombopag before procedures in patients with cirrhosis and thrombocytopenia. *New England Journal of Medicine*.

[25] Afdhal N. H. (2017). Lusutrombopag for treatment of thrombocytopenia in patients with chronic liver disease who are undergoing non-emergency invasive procedures: results from an international phase 3, randomized, double-blind, placebo-controlled study (L-PLUS 2). *Hepatology*.

